# Knowledge, attitudes and practices of South African healthcare workers regarding the prevention and treatment of influenza among HIV-infected individuals

**DOI:** 10.1371/journal.pone.0173983

**Published:** 2017-03-16

**Authors:** Jazmin Duque, Sisanda Gaga, David Clark, Madeleine Muller, Bulenani Kuwane, Cheryl Cohen, Sibongile Walaza, Stefano Tempia, Puleng Ramatoboe, Tsakani Furumele, Marc-Alain Widdowson, Meredith L. McMorrow, Adam L. Cohen

**Affiliations:** 1 U.S. Centers for Disease Control and Prevention, Atlanta, Georgia, United States of America; 2 Battelle Atlanta, Atlanta, Georgia, United States of America; 3 Beyond Zero, Port Elizabeth, South Africa; 4 The Aurum Institute, Johannesburg, South Africa; 5 Centre for Respiratory Disease and Meningitis, National Institute for Communicable Diseases, Johannesburg, South Africa; 6 School of Public Health, Faculty of Health Science, University of Witwatersrand, Johannesburg, South Africa; 7 U.S. Centers for Disease Control and Prevention- South Africa, Pretoria, South Africa; 8 National Department of Health, South Africa, Pretoria, South Africa; 9 United States Public Health Service, Rockville, Maryland, United States of America; Public Health England, UNITED KINGDOM

## Abstract

**Background:**

The South African Department of Health (DOH) publishes annual guidelines identifying priority groups, including immunosuppressed individuals and healthcare workers (HCW), for influenza vaccination and treatment. How these guidelines have impacted HCW and their patients, particularly those infected with HIV, remains unknown.

**Methods:**

We aimed to describe the knowledge, attitudes and practices regarding influenza and the vaccine among South African HCW. Surveys were distributed by two local non-governmental organizations in public health clinics and hospitals in 21 districts/municipalities (5 of 9 provinces).

**Results:**

There were 1164 respondents; median age 41 years; 978/1126 (87%) female; 801/1122 (71%) nurses. One-third (34%) of HCW reported getting influenza vaccine 2013/2014 and most (94%) recommended influenza vaccine to patients infected with HIV. Ability to get vaccine free of charge (aOR 1.69; 95% CI 1.21–2.37) and having received influenza government training (aOR 1.50; 95% CI 1.04–2.15) were significantly associated with self-reported vaccination in 2013/2014. Self-reported 2013/2014 vaccination (aOR 3.76; 95% CI 1.28–11.03) and availability of influenza vaccine during the healthcare visit (aOR 2.56; 95% CI 1.18–5.57) were significantly associated with recommending influenza vaccine to patients infected with HIV/AIDS.

**Conclusion:**

Only one-third of participants were vaccinated in 2013–2014 but those who were vaccinated were more likely to recommend vaccination to their patients. Free and close access to influenza vaccine were associated with a higher likelihood of getting vaccinated in 2013/2014. HCW who reported getting the influenza vaccine themselves, had vaccine to offer during the patient consult and were familiar with DOH guidelines/trainings were more likely to recommend vaccine to HIV-infected patients.

## Introduction

Vaccination is the most effective way to prevent influenza disease. In South Africa, influenza circulates mostly during the winter months of June through August [1]. The South African Department of Health (DOH) provides a limited amount of influenza vaccine in the public sector approximately two months prior to the beginning of the influenza season each year. Influenza vaccine is also available in the private sector. Southern Hemisphere trivalent inactivated influenza vaccine is the only influenza vaccine licensed in South Africa (personal communication). Current guidelines in South Africa recommend influenza vaccine be given to persons at high risk of influenza infection and its complications, particularly individuals with underlying medical conditions like HIV infection. This guideline also states that medical and nursing staff caring for high-risk patients should be vaccinated annually [2]. Estimates suggest that influenza vaccine coverage is low in South Africa, with approximately 25 doses distributed per 1,000 population in both the public and private sector although this may be higher among certain groups [3–5].

Some patients, including those with HIV infection, are at increased risk for severe and sometimes fatal consequences of influenza infection. Cohen, *et al*., estimated that HIV-infected adults aged 25–54 years in South Africa are 2.2 (95% confidence interval (CI): 1.0–5.1) times more likely to die from influenza-related complications than adults aged 65 years or greater in the general population of the United States [6, 7]. Healthcare workers play a major role in the prevention of transmission of influenza viruses as well as the preservation of vital infrastructure to treat persons suffering from influenza-related complications [8]. For this reason, we conducted a cross-sectional survey on the knowledge, attitudes and practices of healthcare workers in South Africa about the prevention and treatment of influenza, particularly for HIV-infected individuals.

## Materials and methods

### Ethics statement

This study was a collaboration between Beyond Zero (previously known as the Institutes for Youth Development, South Africa), the Aurum Institute, the National Institute for Communicable Diseases (NICD) and the U.S. Centers for Disease Control and Prevention (CDC). It was approved by the Internal Review Board at the University of the Witwatersrand in Johannesburg (review number M.130335) and CDC (protocol number 6490). We obtained informed written consent from study participants.

### Survey tool

From October 2013 to April 2014, we conducted a cross-sectional survey about influenza prevention and treatment knowledge, attitudes and practices among public sector healthcare workers in South Africa. Before finalizing the survey tool, we conducted a one-day focus group discussion with South African nurses and doctors to address understandings of influenza-related illness, behaviors and health services. The results from the focus group discussions helped us frame the language and questions used in the survey tool. In addition, we reviewed other survey tools used in knowledge, attitudes and practices studies conducted by the Influenza Division, CDC, and discussed lessons learned with investigators to improve the quality of our survey.

The surveys for this study were collected during a period of low influenza circulation, before the influenza season began. Beyond Zero and the Aurum Institute distributed surveys to healthcare workers in public hospitals, clinics and health centers in 5 of 9 South African provinces (Eastern Cape, Gauteng, KwaZulu-Natal, Limpopo, and North West). The target sample size was 500 completed surveys. Responding to the survey was voluntary and answers remained anonymous and confidential. Vaccination status was collected by self-report and medical records were not consulted.

The survey tool was divided into five sections and had 36 questions. The main outcome variables of interest were self-reported influenza vaccination during 2013/2104 and reports of recommending influenza vaccine to HIV-infected patients. Other variables of interest, examined as independent predictors of self-reported influenza vaccination during 2013/2104 were: role in hospital or clinic, access to influenza vaccine, availability of vaccine at work, ability to get vaccine free of charge, having South African health insurance (medical aid), perceived effectiveness of influenza vaccines, awareness of the national influenza vaccination guideline and having received training from the South African government. Variables examined as independent predictors of recommending influenza vaccine to HIV-infected patients were: self-reported influenza vaccination during 2013/2014, availability of influenza vaccine during patient visit, awareness of the national influenza vaccination guideline and having received training from the South African government. We analyzed the relationships between the main outcomes of interest and the predictor variables by bivariate and multivariate analyses. Data analysis was conducted using SAS version 9.3 (SAS Institute, Cary, NC) and Microsoft Access© (Microsoft Corporation, Redmond, WA). Conditional maximum likelihood estimates of odds ratios and 95% confidence intervals were calculated for bivariate analyses. In order to select the most appropriate multiple logistic regression models, we first assessed partial correlations between the outcome variable of interest and each predictor variable, controlling for the effects of the other variables. We chose the final logistic regression models presented herein using the R-squared selection strategy option in PROC REG. Results were considered statistically significant if the associated two-sided p-value was <0.05.

## Results

**Fig 1:**
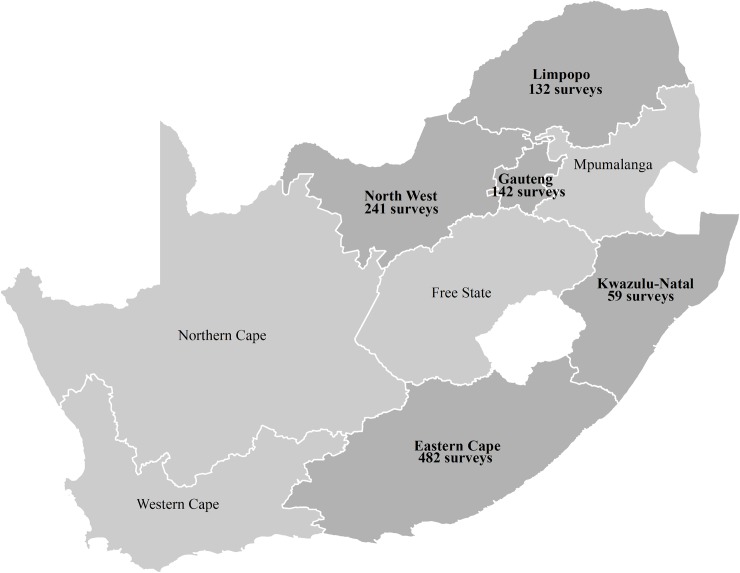
Number of surveys completed by province, South Africa (N = 1056)Ω Ω Missing: province (n = 108).

A total of 1164 surveys were completed although the target sample size was 500. Beyond Zero and the Aurum Institute reported widespread acceptance of the survey. The median age of respondents was 41 years (inter-quartile range: 33–51 years) and 87% were female (Table 1). Of 1122 healthcare workers who responded, occupations included 801 (71%) nurses, 108 (10%) HIV/AIDS counsellors, 76 (7%) community health nurses, 40 (4%) doctors, and 97 (9%) other types of healthcare workers, including 14 pharmacists. The majority of respondents (85%) reported having direct contact with patients seeking HIV/AIDS treatment in their day-to-day responsibilities. Surveys were collected from the following provinces: Eastern Cape (482/1056 [46%]), Gauteng (142 [13%]), KwaZulu Natal (59[6%]), Limpopo (132 [13%]), and North West (241[23%]; Fig 1, S1 Table).

**Table 1:** Characteristics of survey respondents (N = 1164)*.

Characteristic		n (%)
Sex		
	Male	148 (13)
	Female	978 (87)
Highest level of education obtained		
	Certificate or diploma for completing secondary education	645 (63)
	Bachelor's degree	207 (20)
	Professional first degree postgraduate	85 (8)
	General first degree	15 (1)
	Postgraduate degree	31 (3)
	Honours degree	33 (3)
	Master's degree	8 (<1)
	Doctorate degree	7 (<1)
Role in hospital or clinic		
	Community health nurse	76 (7)
	HIV/AIDS counsellor	108 (10)
	Nurse	801 (71)
	Doctor	40 (4)
	Other	97 (9)

*Missing: Sex (n = 38), Province (n = 108), Level of Education (n = 133), Role (n = 42).

Of 1164 respondents, most identified runny nose (94%), fever (92%), cough (87%), and sore throat (78%) as symptoms of influenza illness (Table 2). Regarding causes, 85% of healthcare workers said influenza illness is caused by “a virus, bacteria or germ”, 13% by “cold weather” and <1% by “poor nutrition”. Moreover, 61% said it is possible for a person to be hospitalized because of an influenza illness. When asked to identify high risk groups for influenza illness from a list, respondents correctly circled at least one of these groups 71% to 94% of the time. Of 1130 respondents, 907 (80%) said vaccination should be given annually in order to prevent disease. Most (61%) healthcare workers were aware that there are specific medications designed to treat influenza; however, 25% thought there were none and 14% did not know.

**Table 2:** Knowledge and attitudes of South African healthcare workers regarding influenza infection and vaccination (N = 1164)**.

		n (%)	
What are the symptoms of influenza infection?^Ω^^ϰ^			
	Runny nose	1088 (94)	
	Fever	1070 (92)	
	Cough	1014 (87)	
	Sore throat	908 (78)	
	Chills	861 (74)	
	Difficulty breathing	612 (53)	
	Diarrhea	123 (11)	
What causes influenza?			
	Bacteria, virus or germs	975 (86)	
	Cold weather	157 (13)	
	Poor nutrition	6 (<1)	
Is it possible for a person to be hospitalized because they have the flu?			
	Yes	696 (61)	
	No	442 (39)	
	Don’t know	26 (2)	
Which groups are more likely to have flu-related complications? ^Ω^			
	Persons infected with HIV/AIDS	1094 (94)	
	Persons older than 65	977 (84)	
	Young children	971 (83)	
	Persons with tuberculosis	964 (83)	
	Persons with asthma or other chronic lung disease	952 (82)	
	Persons with chronic medical conditions	907 (78)	
	Pregnant women	875 (75)	
	Healthcare workers	830 (71)	
How effective is influenza vaccine at preventing illness?			
	Highly effective	465 (42)	
	Average	542 (48)	
	Not very effective	84 (8)	
	Not effective	28 (2)	

**Missing: symptoms (N/A), cause (n = 26), die of influenza (n = 51), Groups (N/A), effectiveness (n = 45)

^Ω^Answers were not mutually exclusive

^ϰ^778/1164 (67%) identified all of the following as symptoms of influenza: fever, cough, runny nose and sore throat. 479/1164 (41%) identified all of the previously listed symptoms AND difficulty breathing as symptoms of influenza infection.

Of 1137 healthcare workers, 74% knew that South Africa has national guidelines for seasonal influenza vaccination, and 66% were aware of the national campaign to promote influenza vaccination. Some respondents (19%) reported having received educational training on influenza and influenza vaccines from the South African DOH (Eastern Cape = 65, Gauteng = 27, KwaZulu Natal = 10, Limpopo = 28, North West = 52, missing = 26). Of 1117 respondents, 70% thought that vaccinating healthcare workers helps protect patients against influenza infection. Approximately half (48%) of healthcare workers categorized the effectiveness of influenza vaccines as average when presented with a list of possible categories (Table 2). Protecting themselves, their families and their patients against influenza were reported as more important reasons to get vaccinated than not missing work (p-value <0.01). A majority (81%) of healthcare workers said they have access to influenza vaccines but only 49% reported access to influenza vaccines at their workplace. Moreover, 65% reported access to vaccines free of charge, of which 63% reported having health insurance (medical aid). Overall, 26% of healthcare workers who had medical aid did not know if it covered the cost of getting vaccinated against influenza.

One-third of respondents (34%) reported getting an influenza vaccine in 2013/2014 and 63% reported being vaccinated previously at least once in their life. In bivariate analysis, community healthcare workers (OR 3.47; 95% CI 1.30–10.30), HIV/AIDS counsellors (OR 2.94; 95% CI 1.16–8.41) and nurses (OR 3.11; 95% CI 1.34–8.23) were significantly more likely to get vaccinated in 2013/2014 than medical doctors. Moreover, reported access to influenza vaccine (OR 3.52; 95% CI 2.24–5.71), ability to get vaccine free of charge (OR 1.67; 95% CI 1.24–2.25), and having received influenza government training (OR 1.83; 95% CI 1.33–2.50) were significantly associated with getting vaccinated in 2013/2014. Availability of influenza vaccine at work, having private health insurance (medical aid), opinion of the effectiveness of influenza vaccine, and awareness of national influenza vaccination guideline were not predictors of self-reported influenza vaccination in 2013/2014. Ability to get vaccine free of charge (aOR 1.69; 95% CI 1.21–2.37) and having received influenza government training (aOR 1.50; 95% CI 1.04–2.15) were significantly associated with getting an influenza vaccine in 2013/2014 in a multivariate model adjusting for role in hospital/clinic, access to influenza vaccine, and having private health insurance (Table 3).

**Table 3:** Characteristics among South African healthcare workers vaccinated against influenza in 2013/2014

		Frequency (Row %)		Unadjusted OR		Adjusted OR	
Respondent Characteristics		Vaccinated	Not vaccinated		95% CI		95% CI
Role in hospital or clinic							
	Community health nurse	25 (40)	38 (60)	3.47*	**(1.30–10.30)**	2.90	(0.91–9.28)
	HIV/AIDS counsellor	35 (36)	63 (64)	2.94*	**(1.16–8.41)**	2.70	(0.87–8.43)
	Nurse	277 (37)	475 (63)	3.11*	**(1.34–8.23)**	2.22	(0.85–5.83)
	Doctor	6 (16)	32 (84)	Ref		Ref	
Access to influenza vaccine							
	Yes	348 (40)	533 (60)	3.52*	**(2.24–5.71)**	1.88	(0.84–4.22)
	No	23 (16)	124 (84)	Ref		Ref	
Influenza vaccine available at work							
	Yes	204 (38)	333 (62)	1.29	(1.00–1.66)		
	No	173 (32)	364 (68)	Ref			
Ability to get vaccine free of charge							
	Yes	256 (43)	342 (57)	1.67*	**(1.24–2.25)**	1.69*	**(1.21–2.37)**
	No	90 (31)	201 (69)	Ref		Ref	
Private health insurance (medical aid)							
	Yes	264 (37)	452 (63)	1.27	(0.97–1.66)	1.42	(0.94–2.13)
	No	113 (32)	245 (68)	Ref		Ref	
Opinion of vaccine effectiveness							
	Effective	340 (36)	613 (64)	1.45	(0.93–2.30)		
	Not effective	29 (28)	76 (72)	Ref			
Aware of national guideline							
	Yes	295 (37)	493 (63)	1.35	(0.85–2.20)		
	No	27 (31)	61 (69)	Ref			
Received influenza training from government							
	Yes	92 (47)	105 (53)	1.83*	**(1.33–2.50)**	1.50*	**(1.04–2.15)**
	No	263 (32)	548 (68)	Ref		Ref	

* p-value <0.05.

Most (94%) respondents recommended vaccine to their patients (always = 59%; most of the time = 20%; sometimes = 21%) and 94% of respondents also recommended the vaccine to patients infected with HIV/AIDS (always = 68%; most of the time = 18%; sometimes = 14%). In bivariate analyses, self-reported 2013/2014 vaccination (OR 4.23; 96% CI 2.08–9.63), availability of influenza vaccine during the healthcare visit (OR 3.95; 95% CI 2.14–7.54), awareness of South Africa’s influenza vaccination guidelines (OR 2.82; 95% CI 1.33–5.64), and having received influenza training from the DOH (OR 5.45; 95% CI 1.90–22.14) were associated with a higher likelihood of recommending influenza vaccine to HIV-positive patients. Self-reported 2013/2014 vaccination (aOR 3.76; 95% CI 1.28–11.03) and availability of influenza vaccine during the healthcare visit (aOR 2.56; 95% CI 1.18–5.57) were significantly associated with recommending influenza vaccine to patients infected with HIV/AIDS in a multivariate model adjusting for awareness of the national influenza vaccination guidelines and having received influenza government training (Table 4).

**Table 4:** Characteristics among South African healthcare workers recommending influenza vaccine to HIV-infected patients^±^.

		Frequency (Row %)		Unadjusted OR		Adjusted OR	
Respondent Characteristics		Recommend vaccine	Do not recommend vaccine		95% CI		95% CI
Self-reported 2013/2014 vaccination							
	Yes	359 (98)	8 (2)	4.23*	**(2.08–9.63)**	3.76*	**(1.28–11.03)**
	No	612 (91)	58 (9)	Ref		Ref	
Vaccine available to patients during visit							
	Yes	598 (98)	15 (2)	3.95*	**(2.14–7.54)**	2.56*	**(1.18–5.57)**
	No	343 (91)	34 (9)	Ref		Ref	
Aware of national guideline							
	Yes	775 (96)	36 (4)	2.82*	**(1.33–5.64)**	1.41	(0.54–3.65)
	No	84 (88)	11 (12)	Ref		Ref	
Received influenza training from government							
	Yes	198 (99)	3 (1)	5.45*	**(1.90–22.14)**	2.26	(0.66–7.70)
	No	774 (92)	64 (8)	Ref		Ref	

^±^Recommend always (n = 702), most of the time (n = 188) or sometimes (n = 147)

*p-value <0.05.

## Discussion

Healthcare workers in South Africa may face challenges when recommending influenza vaccine to their patients, even when they are highly trained and when vaccines are available. A study in Kenya found that children were less likely to get vaccinated against influenza if they were younger than 2 years, lived farther than 5 kilometers from a health facility or if their mother was younger than 25 years [9]. It is important to keep in mind that well trained healthcare workers may still face community-level barriers and that vaccine uptake also depends on the willingness of the patient. It is worth noting that this study found that 94% of healthcare workers report recommending vaccine at least some of the time to their patients yet coverage was remarkably low [7, 10].

Our findings are limited to HCP in the public sector. In March 2013, a shorter version of this survey was distributed to mostly private-sector primary healthcare providers participating in the Viral Watch Programme (VWP) which was established to describe influenza seasonality and circulating strains in South Africa. McAnerney et al. found that 98% of VWP healthcare providers recommend influenza vaccine to high-risk patients [5], compared to 94% in our study. Vaccine uptake was much higher amongst the VWP group, with 87/96 (91%) providers reporting getting an influenza vaccine for the current season compared to 34% in our study. Because of their association with the VWP, participants in this study may not have been representative of other private sector practitioners. Although Beyond Zero and the Aurum Institute reported widespread acceptance of the survey, we do not know how many surveys were distributed or the response rate for this study. Moreover, we do not know how representative the study participants are in relation to the South African healthcare worker community at large.

This study was designed to determine the knowledge, attitudes and practices related to influenza illness and influenza vaccination in public healthcare settings in South Africa. We reached 5 of 9 provinces and enrolled a sizeable group of healthcare professionals representing a varied spectrum of education levels. The DOH publishes annual guidelines identifying priority groups for influenza vaccination and treatment. Similar to healthcare workers in Europe and the United States, healthcare workers in South Africa reported that they get vaccinated against influenza primarily to protect themselves, their families and their patients [11, 12]. Messaging focused on how vaccines and antivirals protect healthcare workers and those they come in contact with may increase vaccine uptake. Most importantly, we learned that healthcare workers who have been vaccinated in the past or know a place where they can get vaccine, are much more likely to get vaccinated. As has been found in other studies, increasing availability of vaccines may be the best way to increase vaccination rates and thereby prevent influenza illness in South Africa.

## Conclusions

Most healthcare workers in South Africa surveyed were aware of the causes of influenza illness and ways to prevent it. There was a general understanding of the disease although one in three respondents seemed unaware that influenza could cause severe illness requiring hospitalization and death. Approximately half of the respondents surveyed reported ever being vaccinated against influenza but only one-third reported vaccination in 2013/2014. Role in the hospital/clinic, access to vaccine, ability to get vaccine free of charge and training on influenza were associated with a higher likelihood of getting vaccinated in the current season. Almost every healthcare worker surveyed routinely recommended influenza vaccine to their patients with and without HIV infection, especially when vaccine was available during the healthcare visit. Self-reported vaccination, awareness of the national guideline and influenza training offered by the DOH were also associated with a higher likelihood of recommending influenza vaccine to HIV-infected patients. Knowledge regarding treatment of influenza, specifically antivirals, was less widespread among healthcare workers. Although healthcare workers in South Africa had good knowledge of influenza and influenza vaccination, efforts should be made to increase awareness of the potential severity of influenza disease and to increase vaccination among healthcare workers. Disseminating information to local public health officials in contact with healthcare personnel and using posters in clinics may prove to be effective methods to address this gap.

## Supporting information

S1 Table Knowledge, attitudes and practices of South African healthcare workers regarding the prevention and treatment of influenza among HIV-infected individuals survey responses. (XLSX)
